# The association between cholesterol/saturated fat index (CSI) and quality of sleep, and circadian rhythm among overweight and obese women: a cross-sectional study

**DOI:** 10.1186/s41043-023-00414-1

**Published:** 2023-07-27

**Authors:** Niloufar Rasaei, Mahsa Samadi, Alireza Khadem, Negin Badrooj, Mohadeseh Hassan zadeh, Rasool Ghaffarian-Ensaf, Fatemeh Gholami, Khadijeh Mirzaei

**Affiliations:** 1 Department of Community Nutrition, School of Nutritional Sciences and Dietetics, Tehran University of Medical Sciences (TUMS), P.O. Box: 14155-6117, Tehran, Iran; 2 Department of Nutrition, Science and Research Branch, Islamic Azad University, Tehran, Iran; 3 Department of Nutrition, Faculty of Medicine, Mashhad University of Medical Sciences, Mashhad, Iran

**Keywords:** Circadian rhythms, Obesity, Sleep quality, Woman

## Abstract

**Background:**

The decline in sleep quality is one of the main public health problems affecting the global population. Some studies have shown that a high-fat diet may be linked to changes in circadian rhythm and sleep quality. The cholesterol/saturated fatty acid index (CSI) determines the amount of cholesterol and saturated fatty acid (SFA) in people's dietary patterns and can affect the quality of sleep and circadian rhythm. However, to date, no studies have investigated the effect of this index on these two variables. Therefore, our aim was to investigate the relationship between CSI on circadian rhythm and sleep quality in obese and overweight women.

**Method:**

This cross-sectional study included 378 adult women who were obese or overweight. Using accepted techniques, anthropometric measurements, blood pressure readings, and biochemical variables were evaluated. A validated and trustworthy semi-quantitative food frequency questionnaire (FFQ 147 items) was used to gauge dietary intake. The CSI was measured to find out how much cholesterol and saturated fats were in the diet. Additionally, to assess circadian rhythm and sleep quality, respectively, the valid morning-evening questionnaire (MEQ) and Pittsburgh sleep quality index (PSQI) questionnaires were utilized.

**Result:**

The results of the multinomial logistic regression model of our analysis showed that a significant association was observed between circadian rhythm status with CSI score, and participants with one higher unit of CSI had a 7.3% more chance of being in the eveningness group than being in morningness category in the crude model (OR: 1.07; 95% CI 1.00, 1.14; *P* = 0.026). This association remains marginally significant when adjusting for age, energy intake, BMI, job status, thyroid, and smoking status (OR = 1.08; 95% CI 1.00, 1.16; *P* = 0.051). The binary logistic regression model showed that after controlling for potentially confounding variables, there was no significant association between sleep quality with CSI score, however, those with one higher unit of CSI had 1.6% more chance of having sleep problems (OR: 1.01; 95% CI 0.96, 1.06; *P* = 0.503).

**Conclusion:**

Our results indicated a direct marginally significant association between CSI with evening type in overweight and obese women. Future studies are needed to clarify the precise link between circadian rhythm and sleep behavior with fatty acid quality index.

## Introduction

Circadian rhythms are 24-h cycles characterized by physical, mental, and behavioral changes that can affect important body functions such as hormone secretion, eating habits, food digestion, and body temperature [1]. Suprachiasmatic center (SCN) in the hypothalamus creates the circadian rhythm to regulate sleep and other biological processes [2, 3]. One of the most important circadian rhythms is the sleep–wake cycle [4] and from this point of view, people can be divided into different chronotypes: morningness, intermediate, and eveningness [5]. Morningness people are early risers and have better mental and physical performance in the morning hours, while eveningness people sleep later at night and wake up later in the morning and have their best performance in the afternoon or evening hours [6]. There are also “neither” types who have a moderate intermediate rhythm [7].

By setting the circadian rhythm correctly, the quality of sleep is enhanced, but the changes in this rhythm can affect the quality of sleep and lead to significant disorders such as the inability to fall asleep, disruption of sufficient sleep duration, and difficulty in waking up at the right time [8]. Good sleep quality is directly related to physical, mental, and emotional health, but studies show that poor sleep quality in the long term is associated with increased obesity, type 2 diabetes, and heart disease [9].

Evidence shows that sleep disorders can affect the concentration of lipid profiles in plasma such as triglycerides, cholesterol, and lipoproteins [10, 11]; however, the basic mechanisms of lipid profile abnormalities associated with genes involved in circadian rhythm and sleep duration are not fully understood [12]. The circadian rhythm and sleep quality can also be affected by a high-fat diet [13–15]. In humans, circadian rhythm appears to affect cholesterol synthesis, but its effect on cholesterol absorption remains unclear [16].

The CSI was introduced by Connor et al., to calculate the cholesterol and saturated fatty acid contribution of a dietary pattern [17]. A low score on this index indicates a low content of cholesterol and saturated fat in the food pattern [17–19].

In some studies, a significant relationship has been seen between the consumption of saturated fats and the decrease in sleep depth and quality [15, 20–22], as well as the consumption of saturated fats, especially at night and in the evening with low-quality sleep (low slow wave sleep) [23]. In contrast to these findings, According to a small study involving 8 healthy men, a low-fat, high-carbohydrate diet was associated with less quality sleep after two days compared to a high-fat, low-carbohydrate diet [24]. In another study, it was revealed that high intake of low-fat dairy products and high intake of unsaturated fatty acids could improve sleep status [25, 26]. Also, Animal studies have shown that eating a diet high in saturated fat disrupts the relationship between different phases of the circadian rhythm [13, 27–30].

Considering the contradictory results and the lack of sufficient and clear studies regarding the effect of a diet with high cholesterol and saturated fatty acid content on circadian rhythm and sleep quality, we decided to conduct the present study with the aim of investigating the relationship between CSI and sleep quality and circadian rhythm.

## Methods

### Study population

The present cross-sectional study included 378 overweight and obese women who were referred to health centers in Tehran, Iran. Before taking part in the study, every subject gave their informed consent. Participants were 18–68 years old, with a body mass index (BMI) of 25–39.9 kg/m^2^, with the following exclusion criteria: pregnant or menopause women, lactation, smoking, dieting during the last year, weight loss supplementation, antipsychotic, antihypertensive or lowering glucose, and lipid medications, and women with malignancies; depression; all types of diabetes; liver, kidney, or cardiovascular diseases; and any other acute or chronic diseases. The current study (assigned number: IR.TUMS.VCR.REC.1399.636) was authorized by the Tehran University of Medical Sciences (TUMS) Ethics Committee.

### Anthropometric and blood pressure assessment

We measured anthropometric measurements with a bioelectrical impedance analyzer (BIA), including weight, BMI, fat-free mass (FFM), and body fat percent (BF%); following the manufacturer's protocol (InBody 770 scanner from InBody Co. (Seoul, Korea)) [31]. During the screening, women were asked to remove extra clothing and metal objects such as watches, rings, earrings, shoes, sweaters, and coats. Height was measured using a non-stretch tape measure in a standing-up position barefoot and 0.5 cm of precision. Additionally, the buttocks' narrowest and widest points were used to measure the waist circumference (WC) and hip circumference (HC) with a precision of 0.5 cm. A waist-to-hip ratio (WHR) was calculated by dividing the WC by the HC.

Moreover, blood pressure was measured two times after 5 min of rest using an appropriate cuff according to arms size. Finally, the average of two measurements was reported.

### Biochemical and hormonal determination

After fasting overnight, venous blood was collected. Following centrifugation, serum samples were stored at − 80 °C. The Nutrition and Biochemistry Laboratory of the School of Nutrition and Dietetics at TUMS assessed all samples using standard methods. The enzymatic tests used in this investigation to evaluate fasting blood sugar (FBS), triglycerides (TG), and total cholesterol (TC) were glucose oxidase-phenol 4-amino antipyrine peroxidase (GOD-PAP) and glycerol-3-phosphate oxidase-phenol 4-amino antipyrine peroxidase (GPOPAP). The levels of low-density lipoprotein (LDL) and high-density lipoprotein (HDL) cholesterol were assessed using a direct enzymatic clearance test. Detectable insulin levels were 1.76 mIU/mL, with intra-CV and inter-CV values of 2.19% and 4.4%, respectively. The homeostatic model assessment for insulin resistance (HOMA-IR) was calculated as follows: [(fasting plasma glucose + fasting serum insulin)/22.5] [32]. Standard protocols were used to measure c-reactive protein high-sensitivity (Hs-CRP). All measurements were performed using a Randox Laboratories (Hitachi 902) kit. Liver enzymes including glutamic oxaloacetic transaminase (GOT) and glutamic pyruvic transaminase (GPT) were measured using the Enzymatic Endpoint technique.

### Circadian rhythm and quality of sleep

The validated MEQ was used to determine the circadian rhythms of participants producing peak alertness in the morning or evening. There are a few distinct options and specific scoring options on the questionnaire, which assesses a person's mood on a daily by asking how much sleep they receive and how much time they spend awake, as well as their preferences for physical activity and mental work. Depending on the creators' original analysis, certain options have been given a higher value than others in the questionnaire's alternatives. Typically, the range of scores is 16–86, with the higher scores indicating more time spent in the morning, and lower scores indicating more time spent in the evening. MEQ's basic version splits respondents into five groups depending on their scores: (1) completely morning: 70–86; (2) relatively morning: 59–69; (3) moderate: 42–58; (4) relatively evening: 31–41; and (5) quite evening: 16–30. It has been demonstrated that the MEQ is a valid and reliable tool for measuring circadian rhythms in this population [33]. All of the individuals' Cronbach's alpha coefficients—0.73 for “Morning affect” and 0.80 for “Eveningness”—were satisfactory. The PSQI was used to assess subjective sleep quality. There are 9 items in this questionnaire, but since question 5 contains 10 subitems, the entire questionnaire consists of 19 items that are rated on a 4-point Likert scale from 0 to 3. There are seven subscales in this questionnaire, which are (a) subjective sleep quality, (b) sleep latency, (c) sleep duration, (d) habitual sleep efficiency, (e) sleep disturbances, (f) use of sleeping medications, and (g) daytime dysfunction [34]. PSQI scores are calculated by adding together the seven component scores. An overall score of PSQI ranging from 0 to 21 indicates a lower level of sleep quality [35, 36]. In a previous study [34], the validity and reliability of the Persian version of the PSQI were reported. Cronbach's alpha coefficient for all subjects was 0.77, indicating satisfactory internal consistency.

### Dietary intake assessment

Dietary intake information was collected using a validated and reliable 147-item semi-quantitative FFQ [37]. In the past year, all women recorded their usual frequency of consumption of food items throughout the day, week, and month. An expert dietitian supervised the completion of all FFQs. Using a food analyzer called NUTRITIONIST 4 (First Data Bank, San Bruno, CA), dietary intake was examined for calorie consumption, macronutrients, and micronutrients (gr/day) [38].

### Cholesterol-saturated fat index

Cholesterol and saturated fat concentrations are indicated by CSI. CSI was developed by dividing cholesterol into the saturated fat content of food items [39]. CSI represents a measure of cholesterol and/or saturated fat content, which tells us that a diet with a lower CSI has a lower risk of atherosclerosis and hypocholesterolemia [18, 40].

### Physical activity assessment and other information

A validated and reliable self-report questionnaire, the international physical activity questionnaire (IPAQ), was used to estimate physical activity (PA). The PA level in the last week was measured and presented as metabolic equivalents (MET) [41]. The self-report general questionnaire also assessed employment, education, marriage, and economic status, as well as supplementation intake.

### Statistical analysis

*P* = 0.05 was regarded as statistically significant, whereas *P* = 0.05–0.07 was judged to be marginally significant in the current study. Statistical analyses were carried out using SPSS software (version 26, SPSS Inc., Chicago, IL, USA). Quantitative data were given as means and standard deviation (SD), while categorical data were presented as numbers with percentages. The Kolmogorov–Smirnov test was used to establish the normality of the data distribution. The Chi-square test was used to look into how categorical characteristics (supplement use, educational status, job, income, and marriage) were distributed throughout CSI groups. Analysis of variance (ANOVA) was used to compare the continuous variables such as age, physical activity, anthropometric and body composition measurements, blood pressure, biochemical variables, and dietary intake variables such as food groups, macronutrients, and micronutrients across tertiles of the CSI, along with Bonferroni post hoc analysis when necessary. Estimating energy-adjusted women's food intakes across tertiles of a CSI was done using analysis of covariance (ANCOVA). A multinomial logistic regression model was used to assess the impact of CSI on circadian rhythm status and a binary logistic regression model to assess the impact of CSI on sleep quality.

## Result

### General characteristics of study population according to tertiles of CSI

Table 1 shows the study participants' initial characteristics across CSI tertiles. In the crude model, a significant mean difference was found between CSI tertiles in terms of age (*P* = 0.018) and education (*P* = 0.031), as shown in Table 1. After adjustment with confounders, including BMI, physical activity, and energy intake, the mean difference of education (*P* = 0.009) remained significant and the socioeconomic status (*P* = 0.009) of participants among tertiles of the CSI became significant. Other factors did not significantly differ by mean among CSI tertiles (*P* > 0.05).

**Table 1:** General characteristics of study population according to tertiles of CSI

Variables	CSI				
	Mean ± SD			*P* value*	*P* value_b_
	T1 (*n* = 126)	T2 (*n* = 126)	T3 (*n* = 126)		
Age (years)	37.87 ± 8.95	37.34 ± 9.22	34.81 ± 9.19	**0.018**	0.473
Anthropometric measurements					
Weight (kg)	79.79 ± 10.58	80.12 ± 11.20	81.95 ± 12.10	0.265	0.868
Height (cm)	160.68 ± 5.86	161.27 ± 5.86	161.64 ± 5.63	0.417	0.963
WC (cm)	98.62 ± 9.09	98.73 ± 9.52	100.19 ± 10.09	0.348	0.791
WHR	0.93 ± 0.04	0.93 ± 0.05	0.93 ± 0.05	0.555	0.787
BMI (kg/m^2^)	30.95 ± 3.70	30.76 ± 3.77	31.33 ± 4.07	0.495	0.553
BF (%)	42.03 ± 5.27	41.60 ± 4.95	42.48 ± 5.80	0.427	0.628
FFM (kg)	46.06 ± 5.34	46.48 ± 5.48	46.53 ± 5.77	0.758	0.558
Blood pressure					
SBP (mmHg)	112.88 ± 13.58	111.01 ± 14.02	109.54 ± 12.71	0.280	0.708
DBP (mmHg)	78.52 ± 9.84	77.66 ± 8.49	76.24 ± 10.63	0.314	0.646
Biochemical variables					
FBS (mg/dl)	87.98 ± 10.60	87.14 ± 9.70	86.41 ± 8.23	0.621	0.992
TC (mg/dl)	184.75 ± 31.24	184.22 ± 39.71	181.56 ± 36.17	0.857	0.661
TG (mg/dl)	123.69 ± 81.12	124.06 ± 70.69	113.43 ± 49.64	0.602	0.701
HDL (mg/dl)	47.40 ± 10.41	46.42 ± 12.13	46.09 ± 8.42	0.732	0.874
LDL (mg/dl)	95.08 ± 23.11	92.84 ± 25.15	95.16 ± 23.15	0.779	0.308
GOT (u/l)	16.94 ± 6.28	18.06 ± 7.72	18.53 ± 8.12	0.393	0.447
GPT (u/l)	18.39 ± 12.19	19.23 ± 13.33	19.72 ± 13.70	0.820	0.815
Insulin (mIU/mL)	1.19 ± 0.23	1.22 ± 0.21	1.22 ± 0.23	0.684	0.580
HOMA index	3.42 ± 1.40	3.17 ± 1.17	3.48 ± 1.27	0.269	0.422
hs.CRP (mg/l)	4.58 ± 4.28	5.05 ± 4.75	5.56 ± 4.63	0.230	0.265
Socioeconomic status%(n)				0.103	**0.009**
Poor	50.0 (10)	30.0 (6)	20.0 (4)		
Moderate	38.0 (41)	34.3 (37)	27.8 (30)		
Good	31.4 (59)	29.3 (55)	39.4 (74)		
Education%(n)				**0.031**	**0.009**
Under diploma	52.0 (26)	24.0 (12)	24.0 (12)		
Diploma	30.8 (36)	38.5 (45)	30.8 (36)		
University	30.3 (64)	32.7 (69)	37.0 (78)		
Job%(n)				0.869	0.529
Unemployed	33.6 (73)	34.1 (74)	32.3 (70)		
Employed	32.9 (53)	32.3 (52)	34.8 (56)		
Marriage%(n)				0.155	0.275
Married	34.8 (94)	34.8 (94)	30.4 (82)		
Single	29.6 (32)	29.6 (32)	40.7 (44)		
Supplementation%(n)				0.138	0.142
Yes	30.3 (47)	36.8 (57)	32.9 (51)		
No	38.2 (65)	27.1 (46)	34.7 (59)		

Significant values are in bold

*BF%*—body fat percentage; *BMI*—body mass index; *CSI*—cholesterol to saturated fat index; *DBP*—diastolic blood pressure; *FBS*—fasting blood sugar; *FFM*—fat-free mass; *GOT*—Glutamate oxaloacetate transaminase; *GPT*—glutamate pyruvate transaminase; *HDL*—high-density lipoprotein; *HOMA*—homeostatic model assessment; *hs-CRP*—high-sensitivity C-reactive protein; *SD*—standard deviation; *SBP*—systolic blood pressure; *T*—tertile; *TC*—total cholesterol; *TG*—triglyceride; *WC*—waist circumference; *WHR*—waist-hip ratio

*Calculated by analysis of variance (ANOVA)

*P* value_b_: ANCOVA was performed to adjusted potential confounding factors (energy intake, physical activity, BMI)

Chi-square was used for categorical variables

*P* < 0.05 was considered significant

### Dietary intake of study participants according to tertiles of CSI

Table 2 displays the dietary intakes of the study population by CSI tertile. The participants with the highest tertile of CSI score had significantly higher intakes of energy, carbohydrate, total fat, protein, total fiber, folate, vitamin B-6, and vitamin B12 CSI (*P* < 0.001). After adjustment with the energy intake, there were significant mean differences of carbohydrate, protein, vitamin B-6, vitamin B12, CSI (*P* = 0.001), and total fat (*P* = 0.006) across tertiles of CSI.

**Table 2:** Dietary intake of study participants according to tertiles of CSI

Variables	CSI				
	Mean ± SD			*P* value*	*P* value^b^
	T1 (*n* = 126)	T2 (*n* = 126)	T3 (*n* = 126)		
Nutrient intake					
Energy (kcal/d)	2141.38 ± 670.84	2592.78 ± 696.85	3143.79 ± 725.64	**0.001**	–
Protein (g/d)	67.48 ± 19.00	90.07 ± 21.73	116.48 ± 31.16	**0.001**	**0.001**
Carbohydrate (g/d)	305.72 ± 102.04	385.51 ± 120.18	435.97 ± 96.75	**0.001**	**0.001**
Total fat (g/d)	78.00 ± 31.69	89.47 ± 26.98	116.52 ± 33.07	**0.001**	**0.006**
Vitamin B6 (mg/d)	1.67 ± 0.53	2.21 ± 0.61	2.70 ± 0.74	**0.001**	**0.001**
Folate (mcg/d)	516.36 ± 167.20	624.52 ± 176.13	719.50 ± 181.52	**0.001**	0.301
Vitamin B12 (mcg/d)	2.75 ± 1.04	4.12 ± 1.62	6.19 ± 3.03	**0.001**	**0.001**
Total fiber (g/d)	40.34 ± 20.03	48.03 ± 20.60	54.06 ± 21.80	**0.001**	0.066
CSI	7.86 ± 1.48	12.08 ± 1.25	19.79 ± 4.57	**0.001**	**0.001**
N6/N3	12.72 ± 0.09	12.64 ± 0.09	12.57 ± 0.07	**0.001**	

Significant values are in bold

*CSI*—Cholesterol to saturated fat index; *T*—tertile

*Calculated by analysis of variance (ANOVA)

*P* value^b^: ANCOVA was performed to adjust the potential confounding factor (energy intake)

Data are mean ± SD

*P* < 0.05 was considered significant

### Circadian rhythm status and sleep quality of study participants according to tertiles of CSI

The association between circadian rhythm and sleep quality with tertiles of CSI was shown in Table 3. As shown in the table, there were no significant differences between circadian rhythm and sleep quality with tertiles of CSI (*P* > 0.05).

**Table 3:** Circadian rhythm status and sleep quality of study participants according to tertiles of CSI

Variables		CSI			*P* value
		% (*n*)			
		T1	T2	T3	
*MEQ*	Eveningness	9.8 (11)	15.7 (16)	16.0 (17)	0.204
	Intermediate	57.1 (64)	53.9 (55)	63.2 (67)	
	Morningness	33.0 (37)	30.4 (31)	20.8 (22)	
*PSQI*	Poor sleepers	53.8 (57)	44.9 (44)	54.8 (57)	0.304
	Good sleepers	46.2 (49)	55.1 (54)	45.2 (47)	
	Good sleepers	52.1 (37)	62.9 (44)	51.5 (35)	

*CSI*—Cholesterol to saturated fat index; *MEQ*—morningness-eveningness questionnaire; *PSQI*—Pittsburgh sleep quality index; *SD*—standard deviation; *T*—tertile

*P* < 0.05 was considered significant

### Binary logistic regression model for evaluation of the effect of CSI on sleep quality

The odds ratio and 95% confidence interval for the association between CSI and sleep quality in crude and adjusted models are indicated in Table 4. According to the binary logistic regression model, there was no significant association between sleep quality with CSI score; however, those with one higher unit of CSI had 1.6% more chance of having sleep problems in the crude model (OR:1.01; 95% CI 0.97, 1.05; *P* = 0.432). This association remains non-significant when adjusting for age, energy intake, BMI, job status, thyroid, and smoking status (OR: 1.01; 95% CI 0.96, 1.06; *P* = 0.503) (Table 4).

**Table 4:** Binary logistic regression model for evaluation of the effect of CSI on sleep quality

PSQI	Models			
	Crude		Model 1	
	OR (95%CI)	*P* value	OR (95%CI)	*P* value
Good sleepers	Ref	–	Ref	–
Poor sleepers	1.01 (0.97–1.05)	0.43	1.01 (0.96–1.06)	0.50

*CI*—Confidence Interval; *OR*—odds ratio; *PSQI*—Pittsburgh sleep quality index

*P* values are reported base on the binary logistic regression test

*P* < 0.05 considered as significant

Good sleepers is a reference group

Model 1: Adjusted for age, energy intake, BMI, job status, thyroid and smoking status

### Multinomial logistic regression model for evaluation of the effect of CSI on circadian rhythm status

The multinomial logistic regression model revealed that a significant association was observed between circadian rhythm status with CSI score, and individuals with one higher unit of CSI had 7.3% more chance of being in the eveningness group than being in the morningness category in the crude model (OR: 1.07; 95% CI 1.00, 1.14; *P* = 0.026). This association remains partially significant when adjusting for age, energy intake, BMI, job status, thyroid, and smoking status (OR = 1.08; 95% CI 1.00, 1.16; *P* = 0.051) (Table 5).

**Table 5:** Multinomial logistic regression model for evaluation of the effect of CSI on circadian rhythm status

MEQ	Models			
	Crude		Model 1	
	OR (95%CI)	*P* value	OR (95%CI)	*P* value
Morningness	Ref	–	Ref	–
Intermediate	1.04 (0.99–1.09)	0.092	1.05 (0.99–1.11)	0.087
Eveningness	1.07 (1.00–1.14)	0.026	1.08 (1.00–1.16)	0.051

*CI*—Confidence Interval; *MEQ*—morningness-eveningness questionnaire; *OR*—odds ratio

*P* values are reported base on the multinomial logistic regression test

*P* < 0.05 considered as significant

Morningness is a reference group

Model 1: Adjusted for age, energy intake, BMI, job status, thyroid and smoking status

## Discussion

This study looked at the relationship between the fatty acid quality index (based on CSI tertiles) and sleep quality and circadian rhythm in overweight and obese women. Our findings indicated a positive marginally significant association between greater CSI with being in the eveningness group than the morningness category. However, no strong association was observed with sleep problems.

Given the lack of previous research on the dietary fatty acid quality index (CSI) with sleep quality and circadian rhythm and even less reflected the relationship between high levels of saturated fat intake with sleep problems or circadian rhythm disruption, our results shed light on an unknown association between dietary fat quality measurement with above-mentioned variables.

Three main chronotypes (morning/evening/intermediate) are used to characterize sleep patterns [42]. These three different sleep preferences, based on chronobiology and circadian rhythms [43, 44], are, in part, heritable, but environmental factors including nutrients can modulate chronotype [45]. That is, light–dark cycles influence the central clock, whereas dietary compositions regulate peripheral clocks [46]. The consumption of specific foods and nutrients varied greatly by chronotype, as was previously discussed [47]. We found that participants with higher CSI are more likely to be in the eveningness group. What is less known, though, is to determine whether eveningness leads to elevated CSI or if high CSI alters sleep preference. The correlation between human chronotype and diet has not been widely investigated, and available data have focused on changes in the circadian rhythm on dietary patterns [48]. Fleig and Randler demonstrated that the evening type was linked to higher fat intake [49]. However, there is proof that a high-fat diet can affect the expression of the clock gene both centrally and peripherally, albeit the precise process is still understood [50].

Depending on how sleep is measured, a growing body of literature suggested that the link between fat intake and sleep can vary. Same to our findings, one study among 12 drivers failed to find any relation [51] but one publication on elite female Australian footballers revealed that increased daily saturated fat intake had a significant effect on reduced sleep onset latency (SOL) [52]. Additionally, it has been hypothesized that consuming more saturated fats was linked to a higher risk of the severity of obstructive sleep apnea (OSA) in overweight individuals [53] and shorter sleep duration in a cross-sectional study of 30 healthy Greek women [54]. As reported in the 2007–2008 National Health and Nutrition Examination Survey from 4552 individuals, the cholesterol-rich diet was related to non-restorative sleep [55]. It has been demonstrated that teenagers with emotional eating disorders and night eating syndrome (NES) consume more fat in their diets when their sleep quality is poor [56]. Additional emerging studies mentioned an association between higher fat intake with sleep disorders [57] and lower sleep efficiency (SE), rapid eye movement (REM), and higher slow wave sleep (SWS) [58]. The putative mechanism may be due to the fact that individuals with high dietary fat intake consume low carbohydrates which has been reported to be related to difficulty in maintaining sleep [59].

On the other hand, over and under-consumption of cholesterol have been shown to affect sleep quality and duration negatively [60, 61]. High fatty acid intakes were also found to have positive effects on sleep quality [61–63]. One explanation for the reported association in a study by Lindseth [63] is possibly related to higher levels of docosahexaenoic acid (DHA), thought to increase melatonin concentrations [64] through enzymatic activity required to transform serotonin into melatonin [65]. Melatonin is thought to have a role in a modulating circadian rhythm by weakening the circadian signals and promoting heat loss; thereby improving sleep disturbances and induces sleepiness [66–69]. Furthermore, consuming food and particularly dietary fats stimulates the production of several gut hormones, including cholecystokinin (CCK), which is known to regulate sleep [70]. Sleepiness can be brought on two to three hours after a high-fat meal by the postprandial release of CCK in healthy adults [71]. According to the importance of sleep quality, to examine the precise link between CSI and sleep behavior and to elucidate whether favorable CSI scores can improve sleep disorders and be an adjunct strategy for better sleep quality, further studies are needed.

To our knowledge, this is the first study to assess the effects of CSI changes while targeting both sleep quality and circadian rhythm in overweight and obese women. However, several limitations should be acknowledged. First of all, the cross-sectional study design prevented us clarifying the cause-and-effect relationship between variables. Furthermore, the use of self-reported dietary questionnaire could result in reporting bias. Due to the small number of participants included only overweight and obese women, the current study might not have been adequately powered to detect changes in sleep behaviors and may not be easily extrapolated to all populations. Therefore, additional research on the mediated effect of age on sleep and dietary patterns on a bigger sample and wider spectrum of people is required.

In conclusion, our findings confirm a positive marginally significant association between fatty acid quality measurement with eveningness circadian preference in overweight and obese women. As a result of contradictory conclusions in previous studies, further long-term research is also recommended to evaluate the precise association between circadian rhythm and sleep quality with CSI.

## Data Availability

The data that support the findings of this study are available from correspond author but restrictions apply to the availability of these data, which were used under license for the current study, and so are not publicly available. Data are, however, available from the authors upon reasonable request and with permission of correspond author.

## Abbreviations

ANCOVA: Analysis of covariance
ANOVA: Analysis of variance
BF: Body fat
BIA: Bioelectrical impedance analyzer
BMI: Body mass index
CCK: Cholecystokinin
CSI: Cholesterol/saturated fatty acid index
DHA: Docosahexaenoic acid
FBS: Fasting blood glucose
FFM: Fat-free mass
FFQ: Food frequency questionnaire
GOD-PAP: Glucose oxidase-phenol 4-aminoantipyrine peroxidase
GOT: Glutamic oxaloacetic transaminase
GPO-PAP: Glycerol-3-phosphate oxidase-phenol 4-aminoantipyrine peroxidase
GPT: Glutamic pyruvic transaminase
HC: Hip circumference
HDL: High-density lipoprotein
HOMA-IR: Homeostatic model assessment for insulin resistance
HS-CRP: C-reactive protein high-sensitivity
IPAQ: International physical activity questionnaire
LDL: Low-density lipoprotein
MEQ: Morning-evening questionnaire
MET: Metabolic equivalents
NES: Night eating syndrome
OSA: Obstructive sleep apnea
PA: Physical activity
PSQI: Pittsburgh sleep quality index
REM: Rapid eye movement
SCN: Superchiasmatic center
SFA: Saturated fatty acid
SWS: Slow wave sleep
TC: Total cholesterol
TG: Triglycerides
WC: Waist circumference
WHR: Waist-to-hip ratio

## References

[1] Gumz ML. Circadian clocks: role in health and disease: Springer; 2016.

[2] Allebrandt K, Roenneberg T (2008). The search for circadian clock components in humans: new perspectives for association studies. Braz J Med Biol Res.

[3] Dallmann R, Viola AU, Tarokh L, Cajochen C, Brown SA (2012). The human circadian metabolome. Proc Natl Acad Sci.

[4] Reddy S, Reddy V, Sharma S (2022). Physiology, circadian rhythm.

[5] Hasler BP, Allen JJ, Sbarra DA, Bootzin RR, Bernert RA (2010). Morningness–eveningness and depression: preliminary evidence for the role of the behavioral activation system and positive affect. Psychiatry Res.

[6] Urbán R, Magyaródi T, Rigó A (2011). Morningness–eveningness, chronotypes and health-impairing behaviors in adolescents. Chronobiol Int.

[7] Randler C, Freyth-Weber K, Rahafar A, Jurado AF, Kriegs JO (2016). Morningness–eveningness in a large sample of German adolescents and adults. Heliyon.

[8] Kim JH, Duffy JF (2018). Circadian rhythm sleep–wake disorders in older adults. Sleep Med Clin.

[9] Clement-Carbonell V, Portilla-Tamarit I, Rubio-Aparicio M, Madrid-Valero JJ (2021). Sleep quality, mental and physical health: a differential relationship. Int J Environ Res Public Health.

[10] Chua EC-P, Shui G, Cazenave-Gassiot A, Wenk MR, Gooley JJ (2015). Changes in plasma lipids during exposure to total sleep deprivation. Sleep.

[11] Gangwisch JE, Malaspina D, Babiss LA, Opler MG, Posner K, Shen S (2010). Short sleep duration as a risk factor for hypercholesterolemia: analyses of the national longitudinal study of adolescent health. Sleep.

[12] Xing C, Huang X, Zhang Y, Zhang C, Wang W, Wu L (2020). Sleep disturbance induces increased cholesterol level by NR1D1 mediated CYP7A1 inhibition. Front Genet.

[13] Kohsaka A, Laposky AD, Ramsey KM, Estrada C, Joshu C, Kobayashi Y (2007). High-fat diet disrupts behavioral and molecular circadian rhythms in mice. Cell Metab.

[14] Kim S-M, Neuendorff N, Chapkin RS, Earnest DJ (2016). Role of inflammatory signaling in the differential effects of saturated and poly-unsaturated fatty acids on peripheral circadian clocks. EBioMedicine.

[15] St-Onge MP, Roberts A, Shechter A, Choudhury AR (2016). Fiber and saturated fat are associated with sleep arousals and slow wave sleep. J Clin Sleep Med.

[16] Santosa S, Varady KA, AbuMweis S, Jones PJH (2007). Physiological and therapeutic factors affecting cholesterol metabolism: does a reciprocal relationship between cholesterol absorption and synthesis really exist?. Life Sci.

[17] Connor S, Artaud-Wild S, Classick-Kohn C, Gustafson J, Flavell D, Hatcher L (1986). The cholesterol/saturated-fat index: an indication of the hypercholesterolaemic and atherogenic potential of food. Lancet.

[18] Ghaffarian-Ensaf R, Shiraseb F, Mirzababaei A, Clark CCT, Mirzaei K (2022). Interaction between caveolin-1 polymorphism and dietary fat quality indexes on visceral adiposity index (VAI) and body adiposity index (BAI) among overweight and obese women: a cross-sectional study. BMC Med Genom.

[19] Rasaei N, Daneshzad E, Soveid N, Clark CC, Mirzaei K (2023). The association between fatty acid quality indices and quality of life among overweight and obese women: a cross-sectional study. Front Public Health.

[20] Grandner MA, Kripke DF, Naidoo N, Langer RD (2010). Relationships among dietary nutrients and subjective sleep, objective sleep, and napping in women. Sleep Med.

[21] Kräuchi K, Cajochen C, Werth E, Wirz-Justice A (2002). Alteration of internal circadian phase relationships after morning versus evening carbohydrate-rich meals in humans. J Biol Rhythms.

[22] Hajmir MM, Shiraseb F, Ebrahimi S, Noori S, Ghaffarian-Ensaf R, Mirzaei K (2022). Interaction between ultra-processed food intake and genetic risk score on mental health and sleep quality. Eating Weight Disord Stud Anorex Bulim Obes.

[23] Crispim CA, Zimberg IZ, dos Reis BG, Diniz RM, Tufik S, de Mello MT (2011). Relationship between food intake and sleep pattern in healthy individuals. J Clin Sleep Med.

[24] Phillips F, Crisp A, McGuinness B, Kalucy E, Chen C, Koval J (1975). Isocaloric diet changes and electroencephalographic sleep. Lancet.

[25] Daneshzad E, Heshmati J, Basirat V, Keshavarz SA, Qorbani M, Larijani B (2022). The effect of the dietary approaches to stop hypertension (DASH) diet on sleep, mental health, and hormonal changes: a randomized clinical trial in women with type 2 diabetes. Front Nutr.

[26] Daneshzad E, Mansordehghan M, Larijani B, Heshmati J, Rouzitalab T, Pizarro AB (2022). Diet quality indices are associated with sleep and mental health status among diabetic women: a cross-sectional study. Eating Weight Disord EWD.

[27] Pendergast JS, Branecky KL, Yang W, Ellacott KL, Niswender KD, Yamazaki S (2013). High-fat diet acutely affects circadian organisation and eating behavior. Eur J Neurosci.

[28] Branecky KL, Niswender KD, Pendergast JS (2015). Disruption of daily rhythms by high-fat diet is reversible. PLoS ONE.

[29] Eckel-Mahan KL, Patel VR, De Mateo S, Orozco-Solis R, Ceglia NJ, Sahar S (2013). Reprogramming of the circadian clock by nutritional challenge. Cell.

[30] Lee C, Etchegaray J-P, Cagampang FR, Loudon AS, Reppert SM (2001). Posttranslational mechanisms regulate the mammalian circadian clock. Cell.

[31] Venti CA, Tataranni PA, Salbe AD (2005). Lack of relationship between calcium intake and body size in an obesity-prone population. J Am Diet Assoc.

[32] Mirzaei K, Hossein-Nezhad A, Keshavarz S, Eshaghi S, Koohdani F, Saboor-Yaraghi A (2014). Insulin resistance via modification of PGC1α function identifying a possible preventive role of vitamin D analogues in chronic inflammatory state of obesity. A double blind clinical trial study. Minerva Med.

[33] Rahafar A, Randler C, Díaz-Morales JF, Kasaeian A, Heidari Z (2017). Cross-cultural validity of morningness-eveningness stability scale improved (MESSi) in Iran Spain and Germany. Chronobiol Int.

[34] Farrahi Moghaddam J, Nakhaee N, Sheibani V, Garrusi B, Amirkafi A (2012). Reliability and validity of the Persian version of the Pittsburgh sleep quality index (PSQI-P). Sleep Breath.

[35] Doi Y, Minowa M, Uchiyama M, Okawa M, Kim K, Shibui K (2000). Psychometric assessment of subjective sleep quality using the Japanese version of the Pittsburgh sleep quality index (PSQI-J) in psychiatric disordered and control subjects. Psychiatry Res.

[36] Buysee DJ, Reynolds CF, Monk T, Berman SR, Kupfer DJ (1989). The Pittsburgh sleep quality index: a new instrument for psychiatric practice and research. Psychiatric Res.

[37] Mirmiran P, Esfahani FH, Mehrabi Y, Hedayati M, Azizi F (2010). Reliability and relative validity of an FFQ for nutrients in the Tehran lipid and glucose study. Public Health Nutr.

[38] Ghaffarpour M, Houshiar-Rad A, Kianfar H (1999). The manual for household measures, cooking yields factors and edible portion of foods. Tehran Nashre Olume Keshavarzy.

[39] Mitchell DT, Korslund MK, Brewer BK, Novascone MA (1996). Development and validation of the cholesterol-saturated fat index (CSI) Scorecard: a dietary self-monitoring tool. J Am Diet Assoc.

[40] Rasaei N, Khadem A, Masihi LS, Mirzaei K (2023). Interaction of fatty acid quality indices and genes related to lipid homeostasis on mental health among overweight and obese women. Sci Rep.

[41] Aadahl M, Jørgensen T (2003). Validation of a new self-report instrument for measuring physical activity. Med Sci Sports Exerc.

[42] Roenneberg TKHT, Juda M, Kanterman NT, All Ebrandt K, Gordijn M (2007). Epidem iology of the human circadian clock. Sleep Med Rev.

[43] Tankova I AA B-CG. Circadian typology and individual differences: a review. personality and individual differences. 1994;16:671–684.

[44] Ym KS (2001). Time zones: a comparative genetics of circadian clocks. Natl Rev Genet.

[45] Froy O (2007). The relationship between nutrition and circadian rhythms in mammals. Front Neuroendocrinol.

[46] Rudic RD, McNamara P, Curtis AM (2004). Bmal1 and clock, two essential components of the circadian clock, are involved in glucose homeostasis. PLoS Biol.

[47] Sato-Mito N, Sasaki S, Murakami K, Okubo H, Takahashi Y, Shibata S et al. Freshm en in Dietetic Courses Study II group. The midpoint of sleep is associated with dietary intake and dietary behavi or among young Japanese women. Sleep Med. 2011;12:289–94. 10.1016/j.sleep.2010.09.012 21296614

[48] Beebe DW, Simon S, Summer S, Hemmer S, Strotman D, Dolan LM (2013). Dietary intake following experimentally restricted sleep in adolescents. Sleep.

[49] Fleig DRC (2009). Associ ation between chronotype and diet in adolescents based on food logs. Eat Behav.

[50] Oosterman JEKA, la Fleur SE, Belsham DD (2015). Impact of nutrients on circadian rhythmicity. Am J Physiol Regul Integr Comp Physiol.

[51] Landström UKA, Lennernäs M (2000). Field studies on the effects of food content on wakefulness. Nutr Health.

[52] Condo DLM, Aisbett B, Stevens A, Roberts S (2022). Sleep duration and quality are associated with nutrient intake in elite female athletes. J Sci Med Sport.

[53] Bove CJV, Younes N, Hynes M (2018). What you eat could affect your sleep: dietary findings in patients with newly diagnosed obstructive sleep Apnea. Am J Lifestyle Med.

[54] Rontoyanni VGBS, Cooper AR (2007). Association between nocturnal sleep duration, body fatness, and dietary intake in Greek women. Nutrition.

[55] Grandner MAJN, Gerstner JR, Knutson KL (2014). Sleep symptoms associated with intake of specific dietary nutrients. J Sleep Res.

[56] Ma F (2019). Night eating syndrome and its relationship with emotional eating, sleep quality and nutritional status among adolescents' boys. Community Ment Health J.

[57] Tan XAM, Cheng SM, Mikkola TM, Tenhunen J, Lyytikainen A, Wiklund P, Cong F, Saarinen A, Tarkka I (2015). Associations of disordered sleep with body fat distri bution, physical activ ity and diet among over weight middle-aged men. J Sleep Res.

[58] St-Onge MPMA, Pietrolungo CE (2016). Effects of diet on sleep quality. Adv Nutr.

[59] Tanaka E, Yatsuya H, Uemura M (2013). Associations of protein, fat, and carbohydrate intakes with insomnia symptoms among middle-aged Japanese workers. J Epidemiol.

[60] Gangwisch JEMD, Babiss LA, Opler MG, Posner K, Shen S, Ginsberg HN (2010). Short sleep duration as a risk factor for hypercholesterolemia: analyses of the national longitudinal study of adolescent health. Sleep.

[61] Santana AAPG, Romualdo M, Oyama LM, Santos RVT, Pinho RA, Lira FS (2012). Sleep duration in elderly obese patients correlated negatively with intake fatty. Lipids Health Dis.

[62] Irmisch GSD, Gierow W, Herpertz S, Richter J (2007). Fatty acids and sleep in depressed inpatients. Prostaglandins Leukot Essent Fatty Acids.

[63] Lindseth GMA (2016). Dietary macronutrients and sleep. West J Nurs Res.

[64] Catalá A (2010). The function of very long chain polyunsaturated fatty acids in the pineal gland. Biochem Biophys Acta.

[65] Peuhkuri KSN, Korpela R (2012). Dietary factors and fluctuating levels of melatonin. Food Nutr Res.

[66] Wehr TA, Duncan W, Sher L, Aeschbach D, Schwartz PJ, Turner EH (2001). A circadian signal of change of season in patients with seasonal affective disorder. Arch Gener Psychiatry.

[67] Lundmark PO, Pandi-Perumal SR, Srinivasan V, Cardinali DP, Rosenstein RE (2007). Melatonin in the eye: implications for glaucoma. Exp Eye Res.

[68] Hardeland R. Neurobiology, pathophysiology, and treatment of melatonin deciency and dysfunction. Sci World J. 2012. 10.1100/2012/640389 PMC3354573 22629173

[69] Cajochen C, Kräuchi K, Wirz-Justice A (2003). Role of melatonin in the regulation of human circadian rhythms and sleep. J Neuroendocrinol.

[70] Steiger ADM, Schussler P, Kluge M (2011). Ghrelin in mental health, sleep, memory. Mol Cell Endocrinol.

[71] Wells ASRN, Uvnas-Moberg K, Alster P (1997). Influences of fat and carbohydrate on postprandial sleepiness, mood, and hormones. Physiol Behav.

